# Effect of weight loss intervention on fat metabolism including adipocytokines and enzymes in children with simple obesity

**DOI:** 10.1371/journal.pone.0357145

**Published:** 2026-08-28

**Authors:** Pei-ling He, Yuan-yuan Wang, Shi-han Li, Shi-qiong Wang, Ling-li Sun, Xue-ning Chang, Hai-lin Gu, Xin-yu Ma, Shan-shan Cai, Rui-zhen Li

**Affiliations:** 1 Department of Child Health, Wuhan Children’s Hospital (Wuhan Maternal and Child Healthcare Hospital), Tongji Medical College, Huazhong University of Science and Technology, Wuhan, China; 2 Department of Neonatology, The Second Qilu Hospital of Shandong University, Jinan, China; 3 Department of Radiology, Wuhan Children’s Hospital (Wuhan Maternal and Child Healthcare Hospital), Tongji Medical College, Huazhong University of Science and Technology, Wuhan, China; University of Hawai’i at Manoa College of Tropical Agriculture and Human Resources, UNITED STATES OF AMERICA

## Abstract

**Objective:**

To investigate short-term changes in adipocytokines and fat-metabolism enzymes after a diet and exercise intervention in children with simple obesity.

**Methods:**

Fifty-one children with simple obesity were screened; one did not provide an ELISA serum sample, leaving 50 children with obesity and 50 normal-weight controls for baseline analyses. Forty-four children with obesity completed the 3-month intervention. Leptin, thymic stromal lymphopoietin (TSLP), hormone-sensitive lipase (LIPE/HSL), and carnitine palmitoyltransferase 1A (CPT1A) were measured. Biomarker concentrations were log10-transformed. Baseline comparisons used Welch t tests, repeated measurements used paired t tests, and associations were assessed using Pearson correlation coefficients.

**Results:**

At baseline, children with obesity had higher leptin and lower LIPE/HSL than controls, whereas TSLP and CPT1A did not differ significantly. Among the 44 completers, waist circumference, HDL cholesterol, fasting glucose, 25-hydroxyvitamin D, FT4, and CPT1A decreased, while total cholesterol and leptin increased. Leptin increased and CPT1A decreased in the weight maintenance/gain subgroup; no biomarker change reached statistical significance in the weight-loss subgroup. Baseline leptin was positively correlated with fasting glucose, and baseline CPT1A was positively correlated with triglycerides. Changes in CPT1A, leptin, and TSLP were associated with changes in HDL, insulin, and TSH, respectively.

**Conclusions:**

The 3-month home-based intervention produced a reduction in waist circumference without a significant mean reduction in body weight or BMI. Leptin increased and CPT1A decreased during follow-up, while the participant-level correlation findings were modest and should be considered exploratory.

## Introduction

Childhood obesity has become a major global public health problem and is associated with an increased risk of metabolic, endocrine, cardiovascular, respiratory, and psychosocial complications [1–7]. Although rare monogenic or endocrine disorders may cause obesity in a small proportion of children, most cases are closely related to long-term lifestyle factors, including excessive energy intake, reduced physical activity, and prolonged sedentary behavior [1,2]. Early-onset obesity may persist into adulthood and increases the burden of chronic disease later in life [2,6,7].

Adipose tissue is now recognized as an active endocrine organ. It secretes adipocytokines that participate in energy homeostasis, insulin sensitivity, inflammation, lipid metabolism, and immune regulation [8,9]. Leptin, one of the most extensively studied adipocytokines, is produced mainly by adipocytes and reflects fat mass. Recent reviews continue to support leptin as a potential biomarker of childhood obesity and as an indicator of response to weight-loss interventions [10,11]. However, leptin changes after short-term lifestyle intervention in children remain inconsistent, possibly because leptin is influenced not only by fat mass but also by pubertal development, energy balance, dietary adherence, and the duration and intensity of intervention [10].

Thymic stromal lymphopoietin (TSLP) is an epithelial-derived cytokine involved in immune regulation. Experimental studies have suggested that TSLP may influence adipose loss by promoting sebum-associated lipid excretion, and more recent work has further described TSLP-related IL-4/IL-13 signaling in sebaceous gland activation [12–14]. However, evidence in children with simple obesity is limited.

Fat accumulation reflects the balance between lipid synthesis and lipid catabolism. Hormone-sensitive lipase (LIPE/HSL) and CPT1A are important enzymes involved in lipolysis and mitochondrial fatty acid oxidation [15,16]. CPT1A catalyzes the rate-limiting step of long-chain fatty acid entry into mitochondria for beta-oxidation, and experimental and clinical studies have linked CPT1A and fatty-acid handling to broader metabolic regulation [16–20]. Lifestyle intervention remains a core approach for pediatric obesity management, and recent studies continue to emphasize family-based dietary and physical activity intervention [21,22]. This study therefore examined serum leptin, TSLP, LIPE/HSL, and CPT1A levels in children with simple obesity before and after a 3-month weight loss intervention and explored their associations with anthropometric and metabolic parameters.

## Materials and methods

### Participants and study design

This study was approved by the Ethics Committee of Wuhan Children’s Hospital, Tongji Medical College, Huazhong University of Science and Technology. Written informed consent was obtained from all participating children and their parents or legal guardians before enrollment. Participants were recruited from Wuhan Changchun Street Primary School and the Pediatric Outpatient Department of Wuhan Children’s Hospital between December 1, 2020 and March 31, 2021.

Fifty-one children aged 7–14 years with simple obesity were screened. One child did not provide a serum sample for ELISA and was excluded from the baseline biomarker analysis. The final obesity group comprised 50 children (37 males and 13 females; mean age 10.09 ± 1.23 years). Obesity was defined as BMI at or above the 97th percentile for age and sex according to the International Obesity Task Force criteria [23]. Fifty healthy children of a similar age range were enrolled as normal-weight controls (34 males and 16 females; mean age 10.57 ± 1.12 years). Children were excluded if they had genetic obesity, endocrine obesity, or recent use of medications affecting metabolism, including thyroxine or glucocorticoids.

All 50 children with obesity received the 3-month diet and exercise intervention. Forty-four completed the intervention and follow-up assessments, while six did not complete follow-up. Baseline comparisons included the 50 children with available ELISA measurements. Within-subject analyses included the 44 completers. For consistency with the prespecified archived analysis, the original subgroup allocation was retained: 18 children in the weight-loss subgroup and 26 in the weight maintenance/gain subgroup. One participant lacked anthropometric measurements in the primary follow-up spreadsheet; the archived subgroup assignment was therefore retained for that participant.

### Diet and exercise intervention

The dietary intervention was developed by dietitians and endocrinologists and was implemented at home with monthly professional evaluation. Children with obesity and their parents received nutrition education on obesity, balanced diet, and physical activity. Recommended daily energy intake was 800–1000 kcal/day for children aged 7–10 years and 1000–1200 kcal/day for children aged 10–15 years. The recommended meal distribution was 30% at breakfast, 40% at lunch, and 30% at dinner. The target macronutrient distribution was 40%−45% carbohydrate, 30%−35% protein, and 20%−25% fat, while maintaining adequate intake of protein, vitamins, and minerals.

The exercise intervention included a 5-minute warm-up followed by 30–60 minutes of aerobic exercise, such as brisk walking, jogging, cycling, or swimming, three times per week for 3 months under guardian supervision. Exercise intensity was targeted at 60%−75% of maximum heart rate, calculated as 220 minus age. Sedentary behaviors, including prolonged television viewing and video games, were discouraged.

### Anthropometric and biochemical measurements

Anthropometric and body composition assessments were conducted before and after intervention using an Inbody J30 analyzer. Height, weight, and body fat percentage were measured. Waist circumference (WC) was measured at the midpoint between the iliac crest and the lowest rib. Height was measured to the nearest 0.1 cm and weight to the nearest 0.1 kg with light clothing between 8:30 AM and 10:30 AM by the same evaluator. BMI was calculated as weight (kg)/height (m^2^), and BMI Z-scores were calculated using WHO growth references [24]. Insulin resistance was assessed using HOMA-IR: fasting insulin (mIU/L) × fasting glucose (mmol/L)/ 22.5.

After a 12-hour overnight fast, venous blood samples were collected, centrifuged at 3000 rpm for 15 minutes, and serum was stored at −80 degrees C until analysis. Thyroid-stimulating hormone (TSH), free triiodothyronine (FT3), and free thyroxine (FT4) were measured by direct chemiluminescence. Total cholesterol (TC), triglycerides (TG), low-density lipoprotein (LDL), and high-density lipoprotein (HDL) were analyzed using enzymatic or homogeneous enzyme colorimetry. Leptin (R&D Systems, DLP00), TSLP (Elkbiotech, ELK1083), CPT1A (Elkbiotech, ELK3073), and LIPE/HSL (Elkbiotech, ELK1837) were quantified by ELISA according to the manufacturers’ instructions.

### Statistical analysis

Normality was assessed using the Shapiro-Wilk test. Leptin and TSLP concentrations were transformed as log10(pg/mL). LIPE/HSL and CPT1A values were converted from ng/mL to pg/mL and then log10-transformed before parametric analysis. Continuous variables are presented as mean ± standard deviation. Welch independent-samples t tests were used for baseline comparisons between normal-weight controls and children with obesity, and Fisher exact test was used for sex distribution. Paired t tests were used for within-subject comparisons among the 44 completers and within each prespecified weight-change subgroup. Changes were calculated as post-intervention minus pre-intervention values. Pearson correlation coefficients were calculated between baseline biomarker levels and baseline anthropometric or metabolic variables and between biomarker changes and changes in the corresponding clinical variables. Analyses used pairwise-complete observations, two-sided P < 0.05, and SPSS version 25.0 (IBM Corp., Armonk, NY, USA).

## Results

### Baseline characteristics

The corrected baseline comparison is shown in Table 1. The obesity group included 37 males and 13 females. Children with obesity were slightly younger than controls (P = 0.045) and had higher body weight, waist circumference, BMI, body fat percentage, triglycerides, fasting insulin, HOMA-IR, TSH, FT4, and leptin, together with lower HDL cholesterol, FT3, and LIPE/HSL (all P < 0.05). Height, total cholesterol, LDL cholesterol, fasting glucose, 25-(OH)-D, TSLP, and CPT1A did not differ significantly between groups.

**Table 1:** Baseline anthropometric, biochemical, adipocytokine, and fat-metabolism enzyme characteristics.

Variable	NG (n = 50)	OG baseline (n = 50)	P value
Sex (male/female)	34/16	37/13	0.660
Age at enrollment (years)	10.57 ± 1.12	10.09 ± 1.23	0.045
Height (cm)	146.85 ± 9.23	149.21 ± 9.19	0.202
Body weight (kg)	36.76 ± 7.57	58.16 ± 11.70	<0.001
Waist circumference (cm)	60.37 ± 6.46	86.57 ± 7.38	<0.001
BMI (kg/m²)	16.86 ± 1.82	25.82 ± 2.34	<0.001
Body fat percentage (%)	18.28 ± 5.40	37.62 ± 6.55	<0.001
Triglycerides (mmol/L)	0.72 ± 0.22	1.22 ± 0.62	<0.001
Total cholesterol (mmol/L)	4.02 ± 0.60	3.80 ± 0.80	0.124
HDL cholesterol (mmol/L)	1.64 ± 0.30	1.20 ± 0.23	<0.001
LDL cholesterol (mmol/L)	2.25 ± 0.55	2.38 ± 0.63	0.258
Fasting glucose (mmol/L)	5.14 ± 0.29	5.02 ± 0.40	0.113
Fasting insulin (μIU/mL)	10.25 ± 5.05	21.96 ± 12.64	<0.001
HOMA-IR	2.36 ± 1.23	4.78 ± 2.77	<0.001
25-(OH)-D (ng/mL)	16.17 ± 4.60	15.28 ± 4.32	0.322
TSH	2.27 ± 0.75	2.68 ± 1.09	0.031
FT3	7.04 ± 0.52	6.53 ± 0.52	<0.001
FT4	15.34 ± 1.85	16.45 ± 1.58	0.002
Leptin (log10 pg/mL)	3.34 ± 0.21	3.48 ± 0.17	<0.001
TSLP (log10 pg/mL)	1.99 ± 0.38	2.00 ± 0.40	0.952
LIPE/HSL (log10 pg/mL)	3.57 ± 0.50	3.30 ± 0.44	0.004
CPT1A (log10 pg/mL)	2.20 ± 0.26	2.11 ± 0.34	0.154

Data are mean ± standard deviation or number. Continuous variables were compared using Welch independent-samples t tests; sex distribution was compared using the Fisher exact test. Biomarker values are log10-transformed concentrations. NG, normal-weight group; OG, obesity group; HDL, high-density lipoprotein; LDL, low-density lipoprotein; HOMA-IR, homeostatic model assessment of insulin resistance; TSLP, thymic stromal lymphopoietin; LIPE/HSL, hormone-sensitive lipase; CPT1A, carnitine palmitoyltransferase 1A.

### Overall changes after the 3-month intervention

Among the 44 completers, waist circumference, HDL cholesterol, fasting glucose, 25-(OH)-D, FT4, and CPT1A decreased significantly, whereas total cholesterol and leptin increased significantly (Table 2). Mean body weight, BMI, body fat percentage, triglycerides, LDL cholesterol, fasting insulin, HOMA-IR, TSH, FT3, TSLP, and LIPE/HSL did not change significantly.

**Table 2:** Overall paired pre-post changes among the 44 intervention completers.

Variable	OG-Pre	OG-Post	P value	Paired n
Height (cm)	147.92 ± 7.67	148.71 ± 8.12	0.142	44
Body weight (kg)	56.66 ± 9.84	56.83 ± 9.79	0.482	44
Waist circumference (cm)	85.72 ± 6.60	79.02 ± 7.09	<0.001	42
BMI (kg/m²)	25.70 ± 2.37	25.61 ± 3.07	0.749	44
Body fat percentage (%)	37.63 ± 6.88	37.59 ± 7.58	0.921	43
Triglycerides (mmol/L)	1.29 ± 0.63	1.26 ± 0.54	0.401	43
Total cholesterol (mmol/L)	3.83 ± 0.81	4.03 ± 0.83	0.006	43
HDL cholesterol (mmol/L)	1.21 ± 0.22	1.16 ± 0.22	0.039	43
LDL cholesterol (mmol/L)	2.39 ± 0.65	2.39 ± 0.59	0.941	43
Fasting glucose (mmol/L)	4.96 ± 0.36	4.85 ± 0.36	0.029	43
Fasting insulin (μIU/mL)	22.12 ± 13.14	23.83 ± 14.24	0.324	44
HOMA-IR	4.74 ± 2.85	5.18 ± 3.25	0.288	44
25-(OH)-D (ng/mL)	15.56 ± 4.43	13.28 ± 3.75	<0.001	44
TSH	2.78 ± 1.06	2.83 ± 1.13	0.661	44
FT3	6.52 ± 0.51	6.63 ± 0.51	0.167	44
FT4	16.48 ± 1.59	14.56 ± 1.41	<0.001	44
Leptin (log10 pg/mL)	3.48 ± 0.17	3.63 ± 0.17	<0.001	44
TSLP (log10 pg/mL)	2.01 ± 0.41	1.98 ± 0.22	0.694	44
LIPE/HSL (log10 pg/mL)	3.33 ± 0.43	3.30 ± 0.46	0.539	44
CPT1A (log10 pg/mL)	2.12 ± 0.35	1.88 ± 0.30	<0.001	44

Data are mean ± standard deviation. P values were calculated using paired t tests with pairwise-complete observations; the effective paired sample size is reported for each variable.

### Biomarker changes by weight-change subgroup

The prespecified subgroup results are shown in Table 3. In the weight-loss subgroup, none of the four biomarker changes reached statistical significance; the reduction in CPT1A approached significance (P = 0.057). In the weight maintenance/gain subgroup, leptin increased (P = 0.001) and CPT1A decreased (P = 0.008), whereas TSLP and LIPE/HSL did not change.

**Table 3:** Paired biomarker comparisons within the prespecified weight-change subgroups.

Subgroup	Variable	Pre	Post	P value
Weight loss (n = 18)	Leptin (log10 pg/mL)	3.53 ± 0.19	3.66 ± 0.18	0.074
Weight loss (n = 18)	TSLP (log10 pg/mL)	2.08 ± 0.23	2.02 ± 0.24	0.328
Weight loss (n = 18)	LIPE/HSL (log10 pg/mL)	3.36 ± 0.49	3.28 ± 0.54	0.337
Weight loss (n = 18)	CPT1A (log10 pg/mL)	2.13 ± 0.40	1.95 ± 0.23	0.057
Weight maintenance/gain (n = 26)	Leptin (log10 pg/mL)	3.46 ± 0.16	3.60 ± 0.17	0.001
Weight maintenance/gain (n = 26)	TSLP (log10 pg/mL)	1.95 ± 0.50	1.96 ± 0.21	0.963
Weight maintenance/gain (n = 26)	LIPE/HSL (log10 pg/mL)	3.31 ± 0.39	3.31 ± 0.41	0.972
Weight maintenance/gain (n = 26)	CPT1A (log10 pg/mL)	2.11 ± 0.33	1.83 ± 0.34	0.008

Data are mean ± standard deviation of log10-transformed concentrations. P values were calculated using paired t tests. The archived prespecified subgroup allocation was retained: 18 participants in the weight-loss subgroup and 26 in the weight maintenance/gain subgroup.

### Pearson correlation analysis

Baseline Pearson correlations are presented in Table 4. Leptin was positively correlated with fasting glucose (r = 0.412, P = 0.006), and CPT1A was positively correlated with triglycerides (r = 0.350, P = 0.021). No other baseline associations reached statistical significance.

**Table 4:** Pearson correlations between baseline biomarker levels and baseline anthropometric or metabolic variables.

Variable	Leptin r	P	TSLP r	P	LIPE/HSL r	P	CPT1A r	P
Age (years)	−0.136	0.377	0.142	0.357	−0.033	0.830	0.061	0.692
Body weight (kg)	0.079	0.610	0.010	0.946	−0.169	0.274	−0.119	0.440
Waist circumference (cm)	0.246	0.108	−0.021	0.895	−0.198	0.198	−0.015	0.923
BMI (kg/m²)	0.183	0.235	0.010	0.951	−0.054	0.728	−0.110	0.478
Body fat percentage (%)	0.172	0.265	−0.087	0.576	−0.138	0.373	−0.133	0.390
Triglycerides (mmol/L)	−0.188	0.228	0.063	0.687	−0.020	0.897	0.350	0.021
Total cholesterol (mmol/L)	−0.161	0.301	0.175	0.262	0.096	0.540	0.253	0.102
HDL cholesterol (mmol/L)	−0.086	0.585	0.133	0.397	0.265	0.086	−0.038	0.808
LDL cholesterol (mmol/L)	−0.116	0.458	0.080	0.610	0.052	0.740	0.215	0.167
Fasting glucose (mmol/L)	0.412	0.006	−0.161	0.302	−0.000	0.998	0.091	0.562
Fasting insulin (μIU/mL)	0.040	0.796	0.070	0.654	−0.179	0.246	0.019	0.904
HOMA-IR	0.034	0.824	0.055	0.723	−0.183	0.236	0.053	0.735
25-(OH)-D (ng/mL)	−0.054	0.727	0.221	0.149	0.023	0.880	0.132	0.392
TSH	0.157	0.309	−0.034	0.826	−0.068	0.663	−0.035	0.821
FT3	0.032	0.836	−0.131	0.397	0.094	0.544	0.101	0.515
FT4	−0.179	0.244	−0.021	0.894	0.118	0.444	−0.047	0.762

Values are Pearson correlation coefficients (r) and two-sided P values among the 44 intervention completers. Biomarkers were analyzed after log10 transformation. Pairwise-complete observations were used.

Pearson correlations between changes are shown in Table 5. Change in CPT1A was negatively correlated with change in HDL cholesterol (r = −0.417, P = 0.005), change in leptin was negatively correlated with change in fasting insulin (r = −0.324, P = 0.032), and change in TSLP was positively correlated with change in TSH (r = 0.353, P = 0.019). The remaining change correlations were not statistically significant.

**Table 5:** Pearson correlations between biomarker changes and changes in anthropometric or metabolic variables.

Variable	Leptin r	P	TSLP r	P	LIPE/HSL r	P	CPT1A r	P
Body weight (kg)	0.066	0.671	0.051	0.744	0.006	0.969	−0.110	0.479
Waist circumference (cm)	0.105	0.508	0.296	0.057	0.128	0.419	−0.045	0.779
BMI (kg/m²)	−0.128	0.406	−0.130	0.401	−0.138	0.370	−0.013	0.931
Body fat percentage (%)	0.063	0.687	−0.233	0.132	0.002	0.992	−0.084	0.594
Triglycerides (mmol/L)	−0.132	0.398	−0.056	0.719	−0.114	0.466	0.245	0.113
Total cholesterol (mmol/L)	0.042	0.791	−0.058	0.710	0.153	0.327	−0.249	0.108
HDL cholesterol (mmol/L)	0.016	0.917	−0.028	0.860	0.056	0.720	−0.417	0.005
LDL cholesterol (mmol/L)	0.096	0.542	0.055	0.724	0.232	0.135	−0.229	0.140
Fasting glucose (mmol/L)	0.225	0.147	0.041	0.795	0.061	0.698	0.048	0.761
Fasting insulin (μIU/mL)	−0.324	0.032	−0.006	0.969	−0.108	0.484	0.079	0.610
HOMA-IR	−0.292	0.055	0.000	1.000	−0.159	0.304	0.125	0.418
25-(OH)-D (ng/mL)	−0.022	0.887	−0.079	0.610	−0.088	0.569	0.001	0.994
TSH	−0.073	0.639	0.353	0.019	0.003	0.985	−0.128	0.406
FT3	−0.009	0.954	0.091	0.559	−0.002	0.991	0.130	0.399
FT4	−0.047	0.760	0.143	0.356	0.000	0.998	0.102	0.509

Change was calculated as post-intervention minus pre-intervention. Values are Pearson correlation coefficients (r) and two-sided P values. Biomarkers were analyzed after log10 transformation, and pairwise-complete observations were used.

## Discussion

We rechecked the participant-level clinical and ELISA records before repeating the analyses. In the revised dataset, children with obesity had higher leptin and lower LIPE/HSL than controls, whereas baseline CPT1A was similar between groups. Over the 3-month follow-up, waist circumference decreased, but mean body weight and BMI did not. Leptin rose and CPT1A fell. These findings suggest that circulating adipocytokines and enzymes related to lipid metabolism may change over a short period even when overall body weight remains largely unchanged.

The higher baseline leptin level in the obesity group is consistent with the established relationship between circulating leptin and excess adipose tissue [10,25–29]. The corrected participant-level analysis, however, did not confirm the previously reported correlations with BMI or body fat percentage. Baseline leptin was instead positively associated with fasting glucose, while the change in leptin was inversely associated with the change in insulin. These were moderate associations and cannot establish a causal relationship. Leptin increased in the full follow-up group and in the weight maintenance/gain subgroup, but not in the weight-loss subgroup. The short duration of the intervention, small changes in body fat, differences in adherence, and the timing of blood collection may all have contributed to this pattern [30–35].

CPT1A regulates the entry of long-chain fatty acids into mitochondria for beta-oxidation [16]. After correction of the dataset, baseline CPT1A was not significantly different between groups, but its concentration decreased during follow-up. This decrease was significant in the full follow-up group and in the weight maintenance/gain subgroup; the change in the weight-loss subgroup was close to, but did not reach, statistical significance. Baseline CPT1A was positively associated with triglycerides, and the change in CPT1A was inversely associated with the change in HDL. Serum CPT1A measured by ELISA is not a direct measure of mitochondrial enzyme activity in liver, muscle, or adipose tissue, so these results should be regarded as preliminary observations rather than direct evidence of altered fatty-acid oxidation.

LIPE/HSL was lower in children with obesity at baseline and did not change after the intervention. This may be consistent with altered regulation of lipolysis in obesity, although serum LIPE/HSL does not necessarily reflect intracellular enzyme activity in adipocytes [15,36]. TSLP was similar between groups and remained unchanged during follow-up. Prior experimental and observational studies have linked TSLP to immune regulation, adipose-tissue biology, and metabolic traits [37–41]. We did not expect the positive association between changes in TSLP and TSH. Given the number of exploratory correlations examined, this result may be due to chance and should be confirmed before a biological explanation is proposed.

The intervention did not produce a consistent pattern of metabolic improvement. Waist circumference decreased, whereas body weight, BMI, and body fat percentage showed no significant change. Total cholesterol rose slightly, while HDL, 25-(OH)-D, and FT4 decreased. The reduction in 25-(OH)-D should be interpreted cautiously because vitamin D status in pediatric obesity is influenced by adiposity, metabolic status, and seasonal factors [42–45]. Differences in adherence to the home-based program, pubertal development, seasonal variation, and the relatively short follow-up may have contributed to these mixed findings. In particular, dietary intake and exercise exposure could not be standardized as closely as they would be in a supervised intervention.

Several limitations should be considered. The sample size was modest, the study was not randomized, and only 44 children completed follow-up. Information on pubertal stage, objectively measured physical activity, dietary adherence, and seasonal effects was unavailable. Because some variables had missing values, analyses were based on available pairs. One participant’s follow-up anthropometric measurements were absent from the primary clinical spreadsheet, so the subgroup recorded in the archived prespecified analysis was retained. We also examined multiple exploratory correlations without adjustment for multiple testing. Finally, circulating ELISA concentrations may not reflect enzyme activity in the tissues where lipid metabolism occurs. For these reasons, the findings should be viewed as preliminary.

In this cohort, children with simple obesity had higher leptin and lower LIPE/HSL than normal-weight controls. After 3 months, waist circumference decreased, but mean body weight and BMI did not. Leptin increased and CPT1A decreased, with the clearest changes observed in the weight maintenance/gain subgroup. The participant-level correlations were modest and exploratory. Larger controlled studies with longer follow-up and carefully verified sample tracking are needed to determine whether these biomarker changes are reproducible and clinically meaningful.

## Supporting information

S1 Dataset De-identified clinical, biomarker, paired-analysis, and variable-dictionary data underlying Tables 1–5. (XLSX)
